# Enhanced US/CT/MR imaging of integrin α_v_β_3_ for liver fibrosis staging in rat

**DOI:** 10.3389/fchem.2022.996116

**Published:** 2022-10-03

**Authors:** Xueyao Tang, Xuan Li, Mingxing Li, Xiaoling Zhong, Wenguang Fu, Meng Ao, Jiqing Xuan

**Affiliations:** ^1^ Department of Ultrasound, The Affiliated Hospital of Southwest Medical University, Luzhou, China; ^2^ Department of Ultrasound, The Third People’s Hospital of Chengdu, Clinical College of Southwest Jiao Tong University, The Second Affiliated Chengdu Hospital of Chongqing Medical University, Chengdu, China; ^3^ Department of Ultrasound, Sichuan Provincial People’s Hospital, Chengdu, China; ^4^ Department of Gastroenterology, The Affiliated Hospital of Southwest Medical University, Luzhou, China; ^5^ Academician (Expert) Workstation of Sichuan Province, The Affiliated Hospital of Southwest Medical University, Luzhou, China; ^6^ Department of Hepatobiliary Surgery, The Affiliated Hospital of Southwest Medical University, Luzhou, China; ^7^ Department of Ultrasound, The Second Affiliated Hospital of Chongqing Medical University & Chongqing Key Laboratory of Ultrasound Molecular Imaging, Chongqing, China

**Keywords:** integrin α_v_β_3_, hepatic stellate cell, liver fibrosis, nanoparticles contrast agent, multi-mode molecular imaging

## Abstract

Liver fibrosis is a global health challenge with high morbidity and mortality rates, and diagnostic sensitivity of liver fibrosis tests can be increased using multimodal molecular agents. We designed cyclic arginine-glycine-aspartic acid (cRGD)-modified nanoparticles (NPs) using ultrasound (US)/computed tomography (CT)/magnetic resonance (MR) triple-modality imaging to evaluate liver fibrosis stages. *In vitro* and *in vivo* studies were conducted using primary hepatic stellate cells (HSCs) and a rat model of liver fibrosis induced by carbon tetrachloride (CCl_4_). Our results showed cRGD-poly(lactic-co-glycolic acid)-Fe_3_O_4_-perfluorocarbon bromide (cRGD-PLGA-Fe_3_O_4_-PFOB) NPs were preferentially internalised by activated HSCs (aHSCs). The main cell types expressing integrin α_v_β_3_ during liver fibrogenesis were the aHSCs. The protein levels of α_v_ and β_3_ expressed on aHSCs increased with the progression of liver fibrosis. After intravenous injection of cRGD-PLGA-Fe_3_O_4_-PFOB NPs, the echo intensity (EI) values, CT values, and T2 values of liver parenchyma correlated well with liver fibrosis severity. cRGD-PLGA-Fe_3_O_4_-PFOB NPs as multifunction contrast agents showed great potential to reflect the degree of HSC activation and distinguish among different liver fibrotic stages. The ligand-directed and integrin α_v_β_3_-mediated accumulation provides active and passive targeting capabilities, permitting the targeted multimodal imaging of cRGD-PLGA-Fe_3_O_4_-PFOB NPs, which delivers accurate non-invasive diagnosis and real-time monitoring of liver fibrosis development.

## 1 Introduction

Liver fibrosis, a typical complication of various chronic injuries characterised by excessive synthesis and deposition of extracellular matrix (ECM) proteins, is one of the main causes of mortality and morbidity, affecting over 1,300 million people across the world (Friedman, 2008; Yoon, et al., 2016; Wang, et al., 2014; Williams, 2006). If the underlying aetiologies are eliminated early enough, followed by subsequent aggressive treatments, liver fibrosis may be reversed, otherwise, it will progress to cirrhosis and even liver cancer (Lin, 2017). Such conditions emphasise the urgent need for early detection of liver fibrosis. To date, liver biopsy is the most reliable approach for assessing the severity of liver fibrosis. However, this technique samples a limited liver volume and can only be performed at limited frequencies, out of concern for the incidence of complications (Patel and Rockey, 2006). Clinical biomedical imaging techniques have the potential to enable minimally invasive assessments of liver fibrosis (Cassinotto, et al., 2016; Shin, et al., 2018; Tapper and Loomba, 2018). Clinical techniques hold great promise for anatomical or structural imaging include, but are not limited to, US, CT and MR imaging, whereas their sensitivities are insufficient to detect early liver fibrosis because of their high reliance on hepatic morphological changes (Li et al., 2015; Zhang et al., 2016). Molecular imaging (or functional imaging), which involves the *in vivo* detection of molecular processes, enzymes, and receptors, can detect liver fibrosis at the cellular or molecular level and increase diagnostic sensitivity (Taymouri and Taheri, 2016; Petros and DeSimone, 2010).

Activation of HSCs is a key step in the development of liver fibrosis. In response to various chronic injury factors, the vitronectin receptor integrin α_v_β_3_ drives fibrogenic activation of HSCs (Taymouri and Taheri, 2016). It has been well recognized that the integrin α_v_β_3_ can interact with ECM components via the RGD tri-peptide sequence and the up-regulation of integrin α_v_β_3_ is intimately related to the extent of fibrosis. Consequently, as previous studies demonstrated, a cRGD-based integrin α_v_β_3_ specific molecular probe can be an effective tool for noninvasive assessment of fibrosis progression or therapeutic response (Li, et al., 2011; Zhang, et al., 2017; Xuan, et al., 2017; Li, et al., 2016). Nonetheless, to the best of our knowledge, the majority of such agents only loaded with one imaging material and implemented in a single imaging technique.

PFOB, a liquid perfluorocarbon with a higher density than water, is a viable candidate to be used for multimodal imaging. Owing to their low acoustic velocity and high density, aggregated PFOB microspheres can substantially enhance acoustic reflection (Andre, et al., 1990; Tran, et al., 2007). Moreover, the presence of bromine in the PFOB carbon linear chain allows its detection using CT (Mattrey et al., 1982). Superparamagnetic iron oxide NPs mainly contain iron oxides, Fe_3_O_4_ and Fe_2_O_3_, which have good magnetic sensitivity, and can reduce the transverse relaxation time (Mattrey et al., 1982). In order to improve the independent imaging mode, herein we adopted cRGD-modified PLGA carrying PFOB and Fe_3_O_4_ in our study to get a multimodal US, MR, and CT contrast agent. In present study, we isolated primary rats HSCs to explore the ability of cRGD-PLGA-Fe_3_O_4_-PFOB NPs to target integrin α_v_β_3_ on aHSCs. Further, we compared US/CT/MR molecular imaging results with histological analysis and liver integrin α_v_β_3_ levels to evaluate the feasibility of targeted multimodal molecular imaging as a non-invasive method for early diagnosis and staging liver fibrosis in CCl_4_ rat models.

## 2 Materials and methods

### 2.1 Preparation of cRGD-PLGA-Fe_3_O_4_-PFOB NPs

PLGA-Fe_3_O_4_-PFOB NPs were fabricated using a double emulsion evaporation process. Briefly, 60 μL Fe_3_O_4_, 30 μL PFOB and 50 mg PLGA-PEG-COOH (LA/GA = 50:50, MW = 12,000, Daigang, China) were codissolved in 1 ml of methylene chloride. The solution was emulsified for 4 min using probe sonication (SONICS and MATERALS Inc., Newtown, CT, United States) under 13% output amplitude setting (5 s on and 5 s off). Then, the solution was poured into 5 ml cold poly (vinyl alcohol) (MW = 25,000; Sigma, St. Louis, MO, United States) solution (5% w/v), followed by a secondary emulsification under the same conditions. After adding 10 ml isopropanol solution (2% w/v), the emulsion was magnetically stirred for 6 h (300 rpm, 20–25°C) to extract dichloromethane and isopropanol. PLGA-Fe_3_O_4_-PFOB NPs were obtained after centrifugation at 10,000 rpm and washed three times with deionised water. The steps of cRGD conjugating to PLGA-Fe_3_O_4_-PFOB NPs were the same as our previous study (Xuan, et al., 2017). For confocal microscopy, 50 μL concentrated Nile red solution (0.057 mg/ml in methylene chloride) was added to the organic solution prior to emulsification. The morphological and structural characteristics of NPs were imaged using optical microscopy (CKX41, Olympus, Tokyo, Japan), transmission electron microscopy (TEM, H-7600, Japan), and scanning electron microscopy (SEM, JEM-7800F, Tokyo, Japan). The mean size and zeta potential measurements were performed using a laser particle size analyser system (Zeta SIZER 3000HS: Malvern, United Kingdom). The amount of Fe in the NPs was quantified using atomic absorption spectroscopy.

### 2.2 Detection of conjugation efficiency of cRGD and PLGA-Fe_3_O_4_-PFOB NPs

The Nile Red-labelled PLGA and FITC-labelled cRGD were used for confocal microscopy (CLSM, AIR, Japan) observations. The diluted cRGD-PLGA-Fe_3_O_4_-PFOB NPs were dropped onto a Petri dish and observed under excitation at 543 and 488 nm, respectively. The binding rate of the FITC-labelled cRGD peptide towards PLGA-Fe_3_O_4_-PFOB was further quantitatively evaluated via flow cytometry (FCM, FACS-Calibur, United States) with blank PLGA-Fe_3_O_4_-PFOB NPs as control, and the excitation was set at 488 nm.

### 2.3 *In vitro* cytotoxicity of cRGD-PLGA–Fe_3_O_4_–PFOB NPs

Cell viability was detected using a cell counting kit-8 (CCK-8) cell viability assay (Dongren Chemical & Tech Co., Ltd., China). BRL-3A cells (Procell Life Sci & Tech Co., Ltd., China) were first seeded in a 96-well culture plate at a density of 5×10^3^/well and incubated for 24 h. Then, the medium was switched to a mixed medium containing varying concentrations of cRGD-PLGA-Fe_3_O_4_-PFOB NPs (20, 10, 5, 2.5, 1.25, 0.625, and 0.3125 g/L). Wells without cells or NPs served as blank controls. After 24h, the CCK-8 was added (100 µL) to each well and incubated for 4 h. Finally, the absorbance (A) of each well was measured using a microplate reader (Molecular Devices, United States) at 570 nm.

### 2.4 *In vitro* US/CT/MR imaging

*In vivo* US imaging was performed using a commercial US imaging system (Vevo LAZR, Visual Sonics Inc., Canada). PLGA-Fe_3_O_4_-PFOB NPs aqueous solutions at varying concentrations (50, 25, 12.5, 6.25, and 0 mg/ml) were injected into Eppendorf tubes (EPs, 2 ml). The images were obtained under the same parameter settings (imaging depth = 3.5 cm, mechanical index = 0.1, frequency range: 13–21 MHz). A DFY-type ultrasonic image quantitative analyser (Institution of Ultrasound Imaging of Chongqing Medical University, China) was used to quantify the EI values for each sample. *In vitro* CT imaging was performed using a CT scanner (LightSpeed VCT, GE, United States). EPs filled with diluted suspension were placed in a skull coil and scanned at the same parameter settings: tube current = 180 mA, tube voltage = 110 kV, layer thickness = 0.625 mm. The concentrations of PLGA-Fe_3_O_4_-PFOB NPs suspensions were 50, 25, 12.5, 6.25, and 0 mg/ml, respectively. GE ADW4.4 software was used to measure and compare the CT values of different samples. MR imaging was performed using a clinic 3.0 T MR scanner (Philips, United States). Samples with different iron concentrations (1750, 1,500, 1,250, 1,000, 750, 500, 250, and 0 mg/L) were prepared. Imaging parameters were as follows: repetition time (TR) = 250 ms, echo time (TE) = 15 ms, matrix = 256 mm × 192 mm, slice thickness = 4 mm. The T2 signal intensity values were measured and compared.

### 2.5 Cell-targeting ability of cRGD-PLGA-Fe_3_O_4_-PFOB *in vitro*

HSCs were isolated from rats by collagenase digestion followed by OptiPrep density gradient centrifugation (Sun, et al., 2006; Jeong, et al., 2006; Horiguchi, et al., 2008). After 48 h, primary quiescent HSCs (qHSCs) showed features of myofibroblasts and were therefore identified as aHSCs. Primary rat HSCs cultured for 24 and 48 h were applied for further experiments. After incubation for 30 min with cRGD**-**PLGA**-**Fe_3_O_4_ **-**PFOB NPs (25 mg/ml), cells were washed with phosphate-buffered saline (PBS), fixed in 4% paraformaldehyde, and incubated overnight with primary rabbit glial fibrillary acidic protein (GFAP, diluted 1:200, Abcam, United Kingdom) and primary rabbit *α-*smooth muscle actin (α-SMA, diluted 1:200, Abcam, United Kingdom) at 4°C. The secondary antibody selected for visualisation was FITC-conjugated goat anti-rabbit antibody (1:200; Abcam, United Kingdom). 4′6-diamindino-2-phenylindole (DAPI) was used to dye the cell nuclei. Finally, the ability of cRGD**-**PLGA**-**Fe_3_O_4_ **-**PFOB NPs to target HSCs was observed via CLSM.

### 2.6 Animal models

To establish the liver fibrosis model, eight-week-old female Sprague Dawley rats (200 ± 20 g, Chengdu Dashuo Experimental Animal Co., Ltd., China) were subcutaneously injected with CCl_4_ solution in the back (40% in olive oil, the first dosage: 5 ml/kg, the others: 3 ml/kg) twice weekly for either 3, 6, or 9 weeks (*n* = 12 per group). Controls received normal saline. All experiments were conducted following the guidelines for the care and use of laboratory animals approved by the Ethical Committee of Southwest Medical University.

### 2.7 Biosafety of cRGD-PLGA-Fe_3_O_4_-PFOB NPs

Six normal rats received 50 mg/ml cRGD**-**PLGA**-**Fe_3_O_4_ **-**PFOB NPs at a dose of 5 ml/kg, administered by means of tail vein injection. Serum was collected before and 1 day after injection. Detected biochemical indicators included alanine aminotransferase (ALT), aspartate aminotransferase (AST), total protein (TP), blood urea nitrogen (BUN), creatinine (SCr), andglomerular filtration rate (GFR).

### 2.8 *In vivo* US/CT/MR imaging

Rats with the same administration time were randomly divided into two subgroups. Rats in one group were administered cRGD**-**PLGA**-**Fe_3_O_4_ **-**PFOB NPs while another group received PLGA-Fe_3_O_4_ **-**PFOB NPs. The dose of NPs was set at 5 ml/kg body weight. We first determined an appropriate observed time point by comparing the liver quantitative index of US/CT/MR within 48 h after PLGA**-**Fe_3_O_4_ **-**PFOB NPs injection. All rats were anaesthetised by intraperitoneal injection of 3% pentobarbital sodium at 1 ml/kg. Given the small volume of the rat liver, the right liver parenchyma at the maximum cross-section of the liver was selected as the region of interest (ROI) for quantitative analysis.

*In vivo* US was performed using an ultrasonic diagnostic instrument (Esaote MyLab90, Florence, Italy) in routine B mode. The concentration of NPs was 50 mg/ml. Imaging parameters were as follows: frequency = 10 MHz, mechanical index = 0.1, depth = 44 mm, total gain = 86%. An ultrasonic quantitative analysis diagnostic system (Chongqing Medical University, Chongqing, China) was used to quantitatively measure the EI in the ROI in the liver parenchyma. For CT imaging, the imaging parameters were as follows: tube voltage = 100 kV, tube current = 170 mA, section thickness = 5 mm. The concentration of NPs was 50 mg/mL. GE ADW 4.3 software was used to measure the CT values in the ROI. When rats were subjected to MR imaging, the contrast concentration was adjusted to 10 mg/ml based on the image effect. Liver images were obtained before and after injection using the T2WIM-GRASE sequence. The following parameters were used: TR = 1,100 ms, TE = 20 ms, matrix = 180 × 180; slice thickness = 2 mm; field of view = 120 mm × 120 mm. The T2 values from the T2 map images automatically reconstructed by the system were measured and compared.

### 2.9 Histological analysis of hepatic fibrosis

The livers tissues were fixed in neutralised formalin and embedded in paraffin. The liver sections were stained with haematoxylin and eosin (HE) and Masson’s trichrome and then observed by light microscope (Olympus DP27, Japan). Further semi-quantitative analysis was carried out using image analysis software (Image-Pro Plus 6.0, Media Cybernetics Inc., Silver spring, MD, United States), which could measure the areas of Masson’s trichrome staining (fibrotic).

### 2.10 Immunofluorescence staining

The slices were permeated with 0.5% Triton X-100. Primary antibodies against rat α_v_β_3_ integrin (diluted 1:50, Abcam, United Kingdom), anti-mouse α-SMA antibody (diluted 1:400, Abcam, United Kingdom), and anti-mouse CD31 antibody (diluted 1:50, Abcam, United Kingdom) were used. Secondary antibodies included Alexa Fluor 647-conjugated goat anti-rabbit (diluted 1:400, Abcam, United Kingdom) and FITC-conjugated mouse anti-rat secondary antibodies (diluted 1:400, Abcam, United Kingdom). The liver sections were incubated with mixed primary antibody, mixed secondary antibody, and DAPI. After washing with PBS, the slides were mounted using an anti-fluorescence quencher. Multicoloured fluorescent staining images were analysed via CLSM. The mean fluorescence densities of liver sections were calculated using Image-Pro Plus 6.0. For each group, three amplifying fields (400) were randomly chosen to conduct a semi-quantitative analysis of integrin α_v_β_3_, α-SMA, and CD31 expression levels.

### 2.11 Western blotting

The frozen specimens were lysed in radioimmunoprecipitation assay buffer on ice. After centrifugation, the protein concentration in the supernatant was determined with a bicinchoninic acid protein assay kit. Forty micrograms of total protein were subjected to SDS-PAGE (sodium dodecyl sulfate-polyacrylamide gel electrophoresis) on 10% gels and transferred to a polyvinylidene fluoride membrane. A 5% skimmed milk blocking solution was used to block the cell membrane. Proteins were probed with primary antibodies (α-SMA = 1: 500, αv = 1: 5,000, β3 = 1: 1,000, β-actin = 1: 100,000) overnight at 4°C, followed by horseradish peroxidase-conjugated secondary antibodies. Immunoreactive band quantification was conducted using an enhanced chemiluminescence assay. Glyceraldehyde-3-phosphate dehydrogenase (GAPDH) was used as an internal reference, and the amounts of integrin α_v_, integrin β_3_, and α-SMA protein were determined and expressed as a ratio relative to the GAPDH content.

### 2.12 Statistical analysis

All quantitative data are presented as mean ± standard deviation (SD). Differences were tested using one-way analysis of variance (ANOVA) or Student’s *t*-tests in SPSS statistical package (Version 13.0, SPSS Inc. Chicago, IL, United States). Statistical significance was set at *p* < 0.05.

## 3 Results and Discussion

### 3.1 Characterisation of cRGD-PLGA-Fe_3_O_4_-PFOB NPs

**FIGURE 1:**
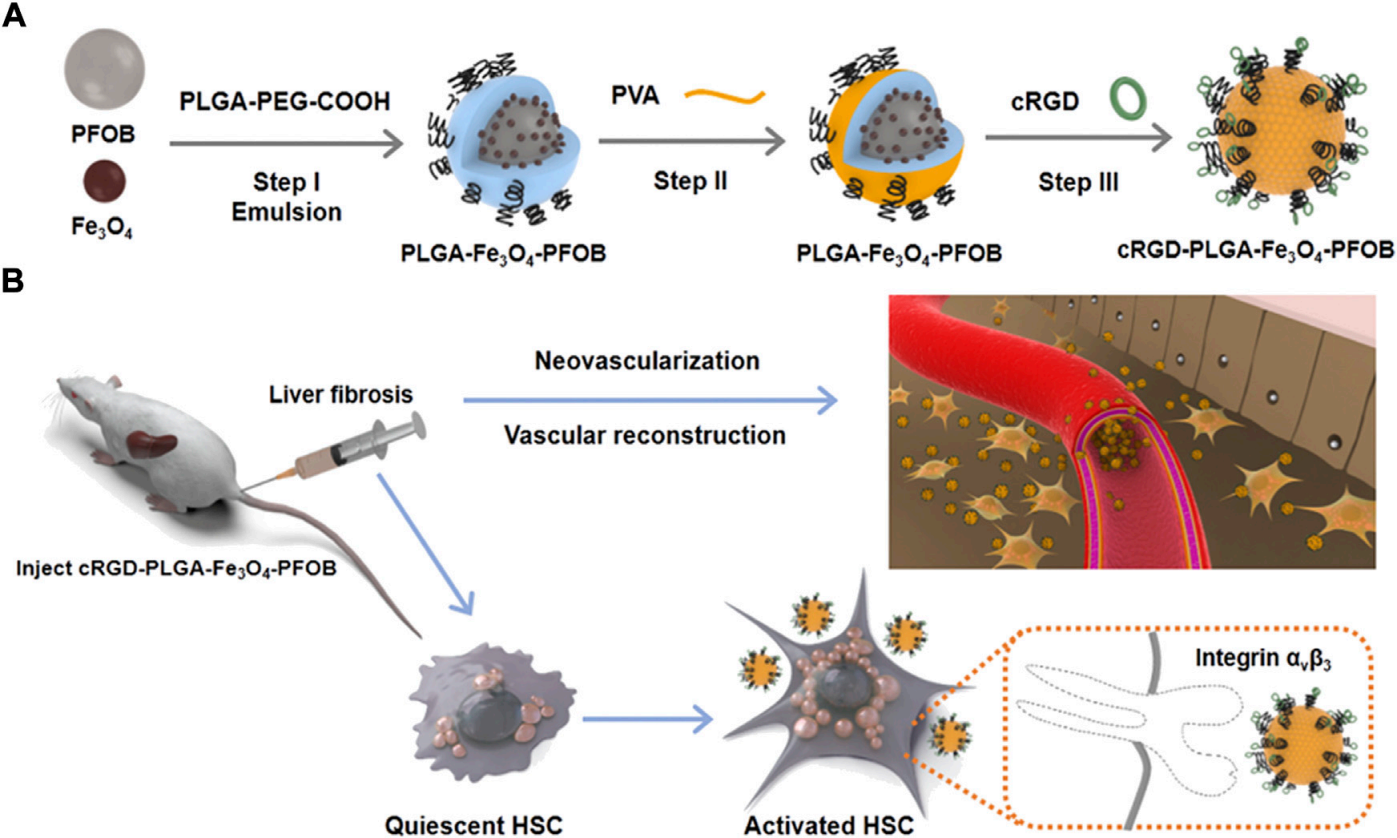
Schematic illustration of fabrication process of **(A)** cRGD-PLGA-Fe_3_O_4_-PFOB NPs and **(B)** their targeting capabilities to integrin α_v_β_3_ expressed by aHSCs.

**FIGURE 2:**
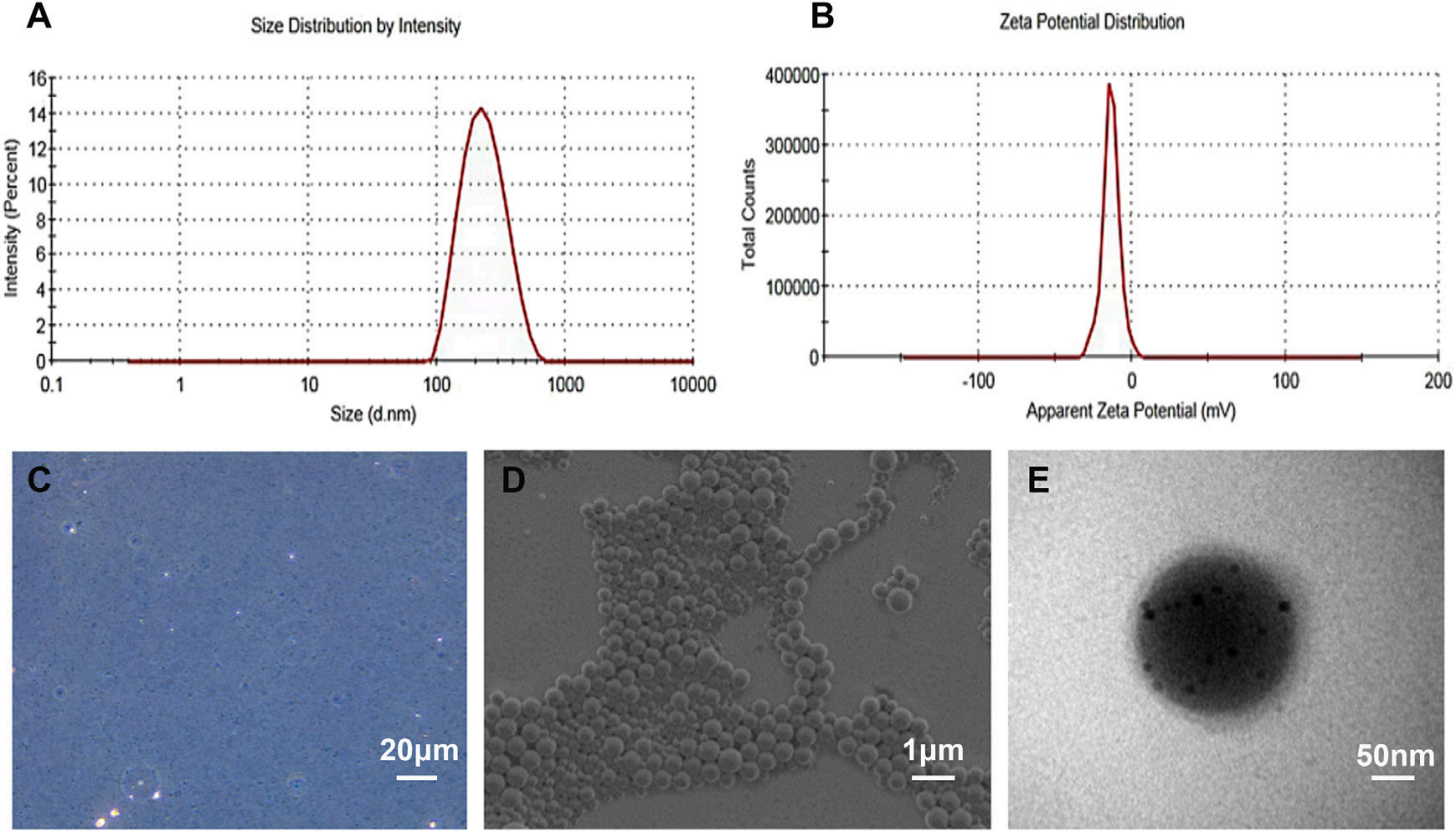
Characterization of the as-prepared NPs. **(A)** DLS size distribution of cRGD-PLGA-Fe_3_O_4_-PFOB NPs. **(B)** The zeta potential of cRGD-PLGA-Fe_3_O_4_-PFOB NPs. **(C)** Light microscopy image of cRGD-PLGA-Fe_3_O_4_-PFOB NPs. **(D)** SEM image of cRGD-PLGA-Fe_3_O_4_-PFOB NPs. **(E)** TEM image of cRGD-PLGA-Fe_3_O_4_-PFOB NPs.

cRGD-PLGA-Fe_3_O_4_-PFOB NPs was prepared as previously reported by Xuan and Dong (Xuan, et al., 2017; Dong, et al., 2019), with slight modifications. Figure 1A shows the schematics of the cRGD-PLGA-Fe_3_O_4_-PFOB NPs. Figure 1B shows the targeting process of the cRGD-PLGA-Fe_3_O_4_-PFOB NPs to integrin α_v_β_3_ *in vivo*. We chose PLGA-PEG-COOH as the carrier. The addition of PEG reduced the recognition by the reticuloendothelial system and the immune system, which prolonged the blood circulation time *in vivo*. On average, all NP preparations were approximately 221 nm in size, and the polydispersity index was below 0.20, verifying size homogeneity (Figure 2A). The zeta potential was −9.3 ± 4.3 mV (Figure 2B). The optical microscope image (Figure 2C) showed that the NPs were small spheres and distributed evenly in the suspension, indicating that they attained uniform size and good dispersion. Dynamic light scattering (DLS) size and optical morphology were verified via SEM (Figure 2D), which revealed a uniform spherical morphology with smooth surfaces. To investigate whether PFOB and Fe_3_O_4_ could be successfully loaded into PLGA, diluted NP suspension samples were observed via TEM. As shown in Figure 2E, the prepared NPs appeared as a core-shell structure with several black iron particles distributed on the shell membrane. Meanwhile, a significant density difference between the shell and the core indicated that PFOB was effectively encapsulated in PLGA NPs. Furthermore, the loading rate of Fe_3_O_4_ was calculated as approximately 38%, according to the atomic absorption spectrometry results.

### 3.2 Conjugation efficiency of cRGD on PLGA-Fe_3_O_4_-PFOB NPs

**FIGURE 3:**
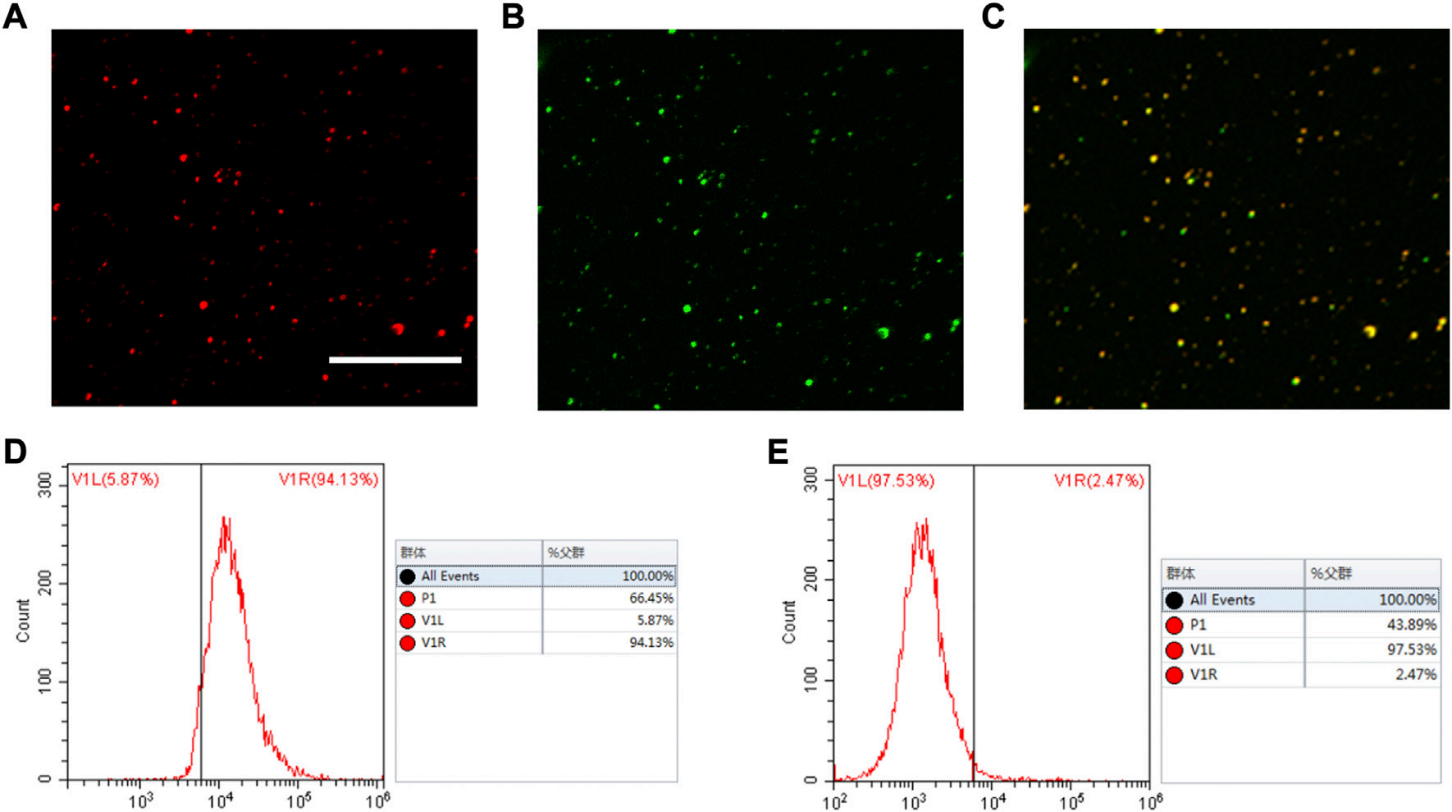
CLSM and FCM images of the cRGD-PLGA-Fe_3_O_4_-PFOB NPs. **(A)** Nile red (×400; Scale: 50 μm). **(B)** FITC. **(C)** Merge. **(D)** FCM image of PLGA-Fe_3_O_4_-PFOB NPs modified with FITC-labelled cRGD peptide. **(E)** FCM image of PLGA-Fe_3_O_4_-PFOB NPs.

The technique used to conjugate cRGD peptides to the PLGA**-**Fe_3_O_4_ **-**PFOB NP surface was derived from the carbodiimide method described in previous reports (Xuan, et al., 2017; Sanna, et al., 2013). This covalent binding mode was stable, effective, and not greatly influenced by the shear flow in blood circulation. To visualise whether cRGD could be successfully connected to PLGA, Nile Red-tagged PLGA**-**Fe_3_O_4_ **-**PFOB NPs and FITC-labelled cRGD were used. The CLSM image shows that the localisation of PLGA**-**Fe_3_O_4_ **-**PFOB NPs (red) largely overlapped with cRGD (green), and the overlapping areas were bright yellow in colour (Figures 3A–C). Next, we performed FCM analysis to further verify the CLSM results. As illustrated in Figure 3D, the FITC positive rate of cRGD-PLGA-Fe_3_O_4_-PFOB NPs was 94.13%. Compared to the NPs without cRGD (only 2.47%, Figure 3E), the FITC positive rate was also significantly higher. Both the CLSM and FCM findings indicated that the cRGD was successfully conjugated onto PLGA-Fe_3_O_4_ **-**PFOB NPs with an excellent connection rate.

### 3.3 *In vitro* US/CT/MR imaging

**FIGURE 4:**
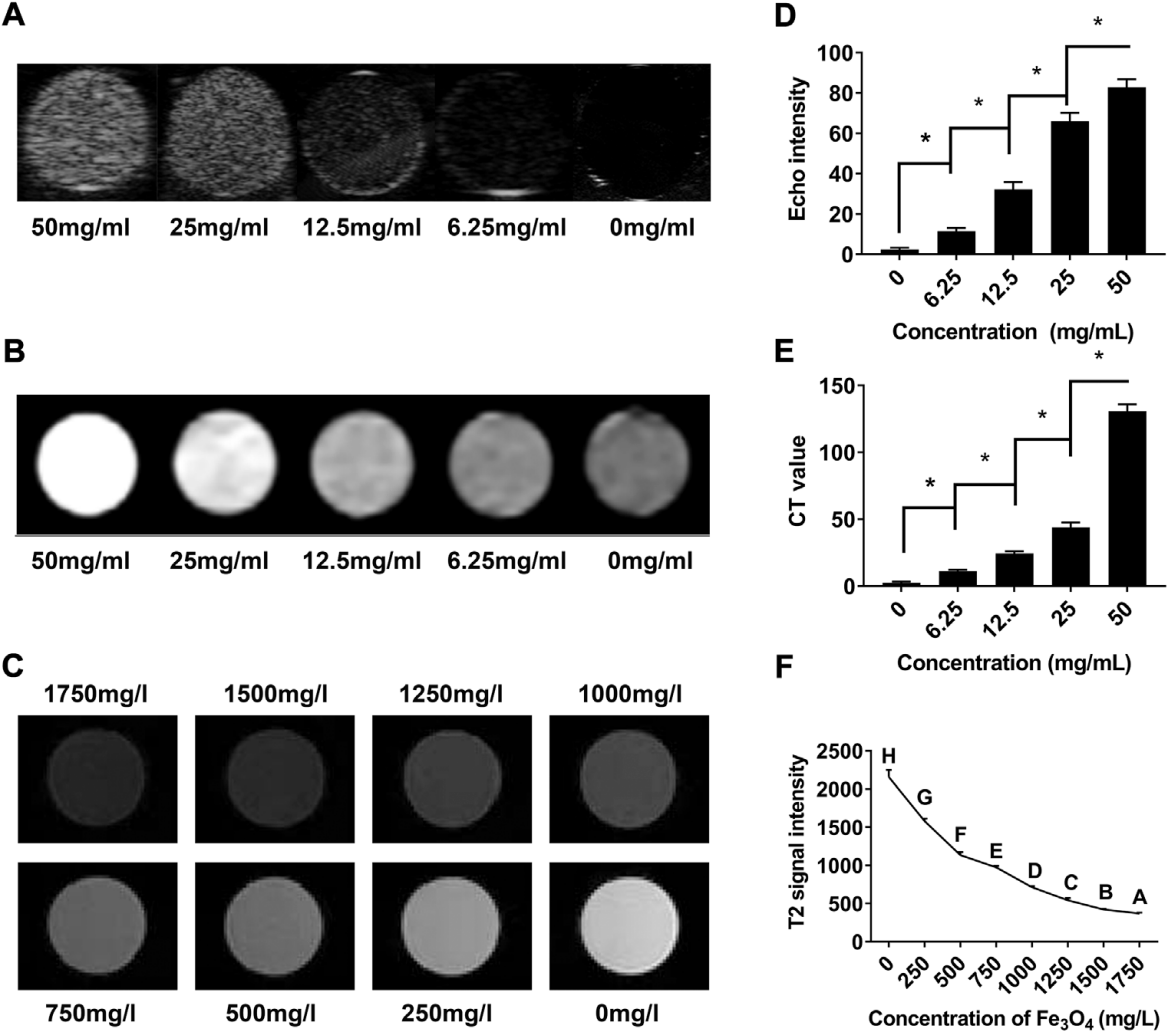
*In vitro* assessment of cRGD-PLGA-Fe_3_O_4_-PFOB NPs. **(A)** US images of cRGD-PLGA-Fe_3_O_4_-PFOB NPs at different concentrations. **(B)** CT images of cRGD-PLGA-Fe_3_O_4_-PFOB NPs at different concentrations. **(C)** T2-weighted MR images of cRGD-PLGA-Fe_3_O_4_-PFOB NPs at different Fe concentrations in water. **(D)** Echo intensity of cRGD-PLGA-Fe_3_O_4_-PFOB NPs at different concentrations. **(E)** CT values of cRGD-PLGA-Fe_3_O_4_-PFOB NPs at different concentrations. **(F)** T2 signal intensity of cRGD-PLGA-Fe_3_O_4_-PFOB NPs at different Fe concentrations in water.

To investigate the US/CT/MR imaging capacities of cRGD**-**PLGA**-**Fe_3_O_4_ **-**PFOB NPs *in vitro*, we first observed the images of EPs containing suspension with different concentrations in an agarose gel model. EPs filled with higher concentrations of NPs suspensions appeared brighter in US and CT images and dimmer in MR images (Figures 4A–C). Quantitative analysis results showed that the EI and CT values increased with increasing NP concentrations, whereas the T2 signal intensity values decreased (Figures 4D–F). These findings confirmed that the cRGD-PLGA-Fe_3_O_4_-PFOB NPs contain the imaging properties of PFOB and Fe_3_O_4_ and have the potential to serve as US/CT/MR multimodality contrast agents for *in vivo* applications.

### 3.4 Cytotoxicity and acute biosafety of cRGD-PLGA-Fe_3_O_4_-PFOB NPs

To verify the cytotoxicity of cRGD**-**PLGA**-**Fe_3_O_4_ **-**PFOB NPs, BRL-3A cells were incubated in cell-culture media, which contain cRGD**-**PLGA**-**Fe_3_O_4_ **-**PFOB NPs. All the treated cells exhibited similar morphologies. The BRL-3A cell viability was measured as (88.69 ± 2.71)% (88.68 ± 4.17)%, (100.01 ± 7.65)% (89.93 ± 5.16)%, (98.23 ± 6.77)% (92.29 ± 4.95)%, and (96.18 ± 8.23)% for groups cultured with cRGD**-**PLGA**-**Fe_3_O_4_ **-**PFOB NPs at concentrations of 20, 10, 5, 2.5, 1.25, 0.625, and 0.3125 mg/ml, respectively. These results did not show a specific correlation between NP concentrations and cell viability, nor did they display a significant difference among NP groups in contrast to the control group (*p* > 0.05), implying that NPs were almost non-toxic over each concentration range. To further verify the acute biosafety of cRGD**-**PLGA**-**Fe_3_O_4_ **-**PFOB NPs *in vivo,* the tail veins of the rats were injected with a dosage of 5 mL/kg from 50 mg/ml NPs suspensions. No significant changes were observed in the serum biochemical indicators (liver and kidney function) 24 h post-injection (Table 1). These results were in good agreement with the cytotoxicity assays, indicating that intravenously injected cRGD-PLGA-Fe_3_O_4_ **-**PFOB NPs are biocompatible.

**TABLE 1:** Serum biochemical indicators post cRGD-PLGA-Fe_3_O_4_-PFOB NPs injection (50 mg/ml).

Indicators	Pre-injection	Post-injection
**ALT (U/L)**	46.53 ± 12.08	52.75 ± 14.39
**AST (U/L)**	122.48 ± 36.02	155.50 ± 40.55
**TP (g/L)**	52.02 ± 4.99	53.45 ± 4.34
**GFR (ml/min)**	218.40 ± 9.54	215.72 ± 12.10
**SCr (μmol/L)**	28.33 ± 2.89	29.32 ± 3.68
**BUN (mmol/L)**	5.33 ± 0.99	5.13 ± 0.30

Note: pairwise comparisons of the same indicators before and after injection (all *p* > 0.05).

### 3.5 Binding characterization of cRGD-PLGA-Fe_3_O_4_-PFOB NPs *in vitro*

**FIGURE 5:**
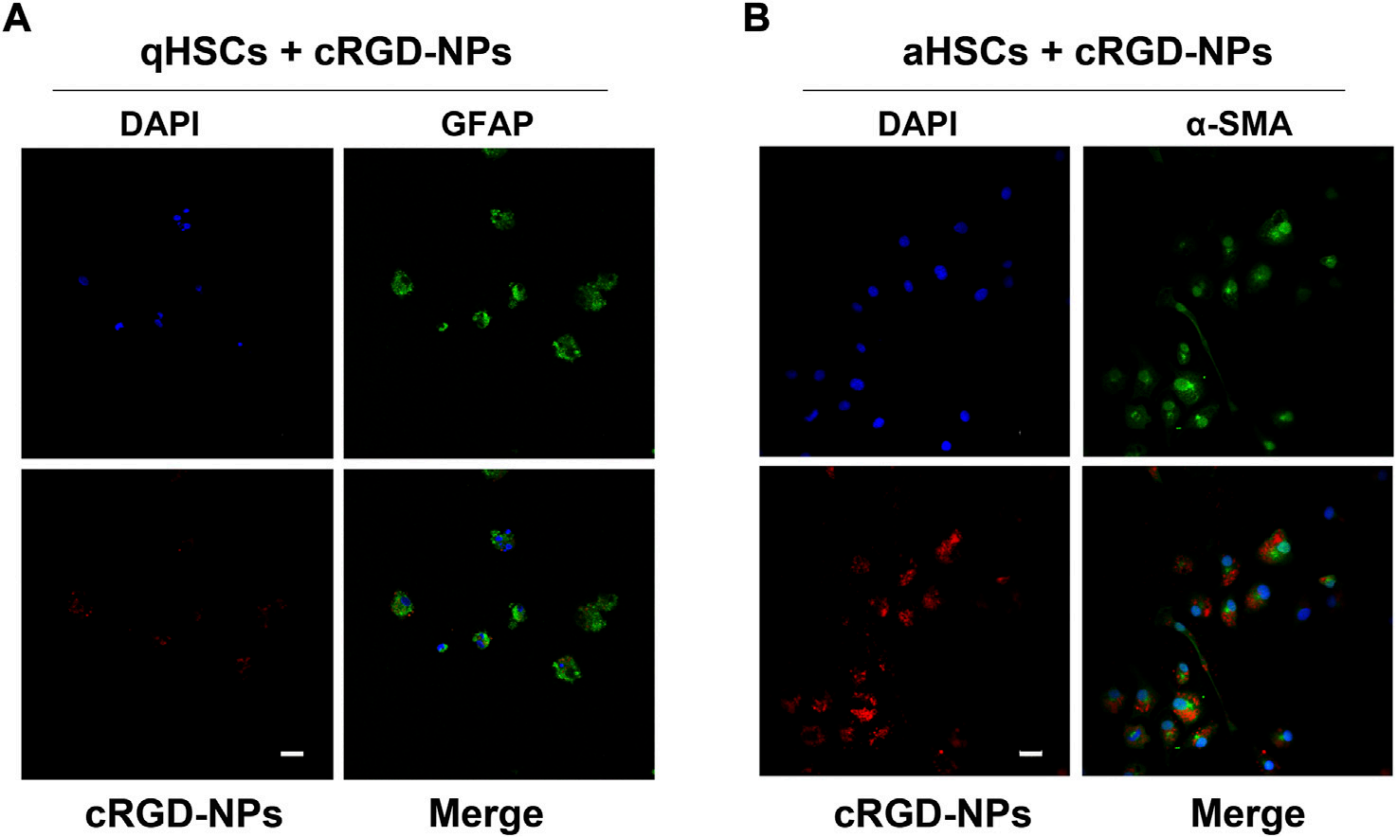
Representative fluorescent images of qHSCs **(A)** and aHSCs **(B)** incubated with 25 mg/ml of cRGD-PLGA-Fe_3_O_4_-PFOB NPs (red) solution for 30 min at 37°C in the dark (×400; Scale: 100 μm).

cRGD peptides are known as one of the most effective functioning elements for delivering diagnostic ‘probes’ into fibrotic livers owing to their rich receptor capacity and prominent receptor-coupling affinity to integrin α_v_β_3_ receptors (Li, et al., 2011; Zhang, et al., 2017; Xuan, et al., 2017). As reported previously (Li, et al., 2011; Zhou, et al., 2004), integrin α_v_β_3_ was highly expressed on aHSC membranes but was found at a very low expression level in qHSCs. To confirm the ability of cRGD**–**PLGA**–**Fe_3_O_4_ **–**PFOB NPs to target aHSCs *in vitro*, we isolated primary HSCs from normal rats. As presented in the CLSM results (Figures 5A,B), aHSCs demonstrated significantly higher uptake of cRGD**–**PLGA**–**Fe_3_O_4_ **–**PFOB NPs compared to qHSCs. Very low uptake of cRGD**–**PLGA**–**Fe_3_O_4_ **–**PFOB NPs in qHSCs may be the result of passive uptake or nonspecific endocytosis. We demonstrated that adding cRGD peptide to NPs causes them to specifically target aHSCs *in vitro*, laying a foundation for targeted imaging *in vivo*.

### 3.6 Liver fibrosis stage analysis

**FIGURE 6:**
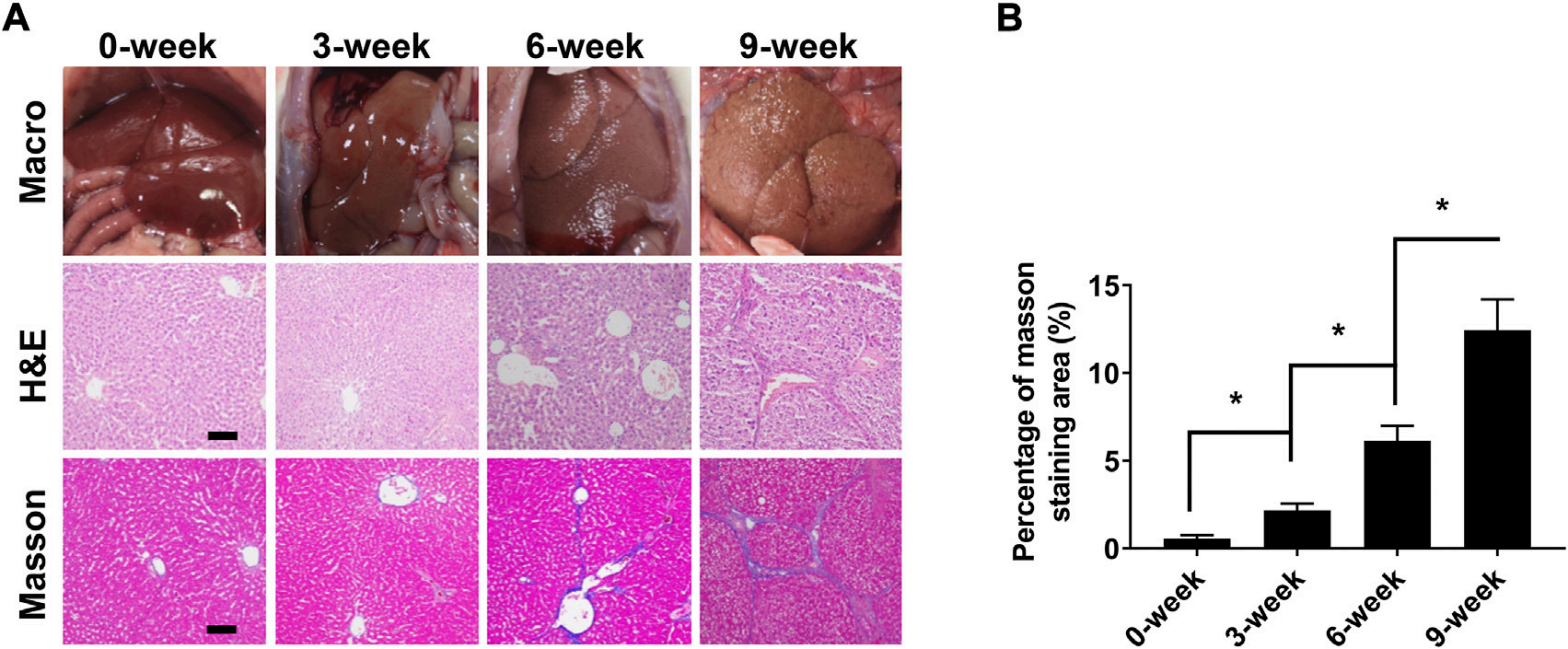
Liver fibrosis stage analysis after 0, 3, 6, and 9 weeks of CCl_4_ induction. **(A)** Macroscopic inspections and pathological examination of the liver fibrosis and control groups (×100; Scale: 100 μm). **(B)** Semi quantitative analysis of Masson’s trichrome staining (fibrosis) in normal and fibrotic livers. *p < 0.05.

During the 9 weeks of CCl_4_ administration, liver fibrosis and disease progression was confirmed by anatomical visualisation upon dissection, histologic staining and areas measurements of collagen deposition (Figures 6A,B). No noticeable fibrosis changes were observed in the livers of the 0-weeks group. With prolonged CCl_4_ administration, the structure of normal liver lobules was damaged, pseudo-lobules appeared, and the fibres surrounding the pseudo-lobules increased and widened significantly. Furthermore, the collagen deposition area increased with the prolonged period of CCl_4_ injection. These results suggested that our animal model could effectively present the pathological progression of liver fibrosis.

### 3.7 Biomarkers expression in fibrotic livers

**FIGURE 7:**
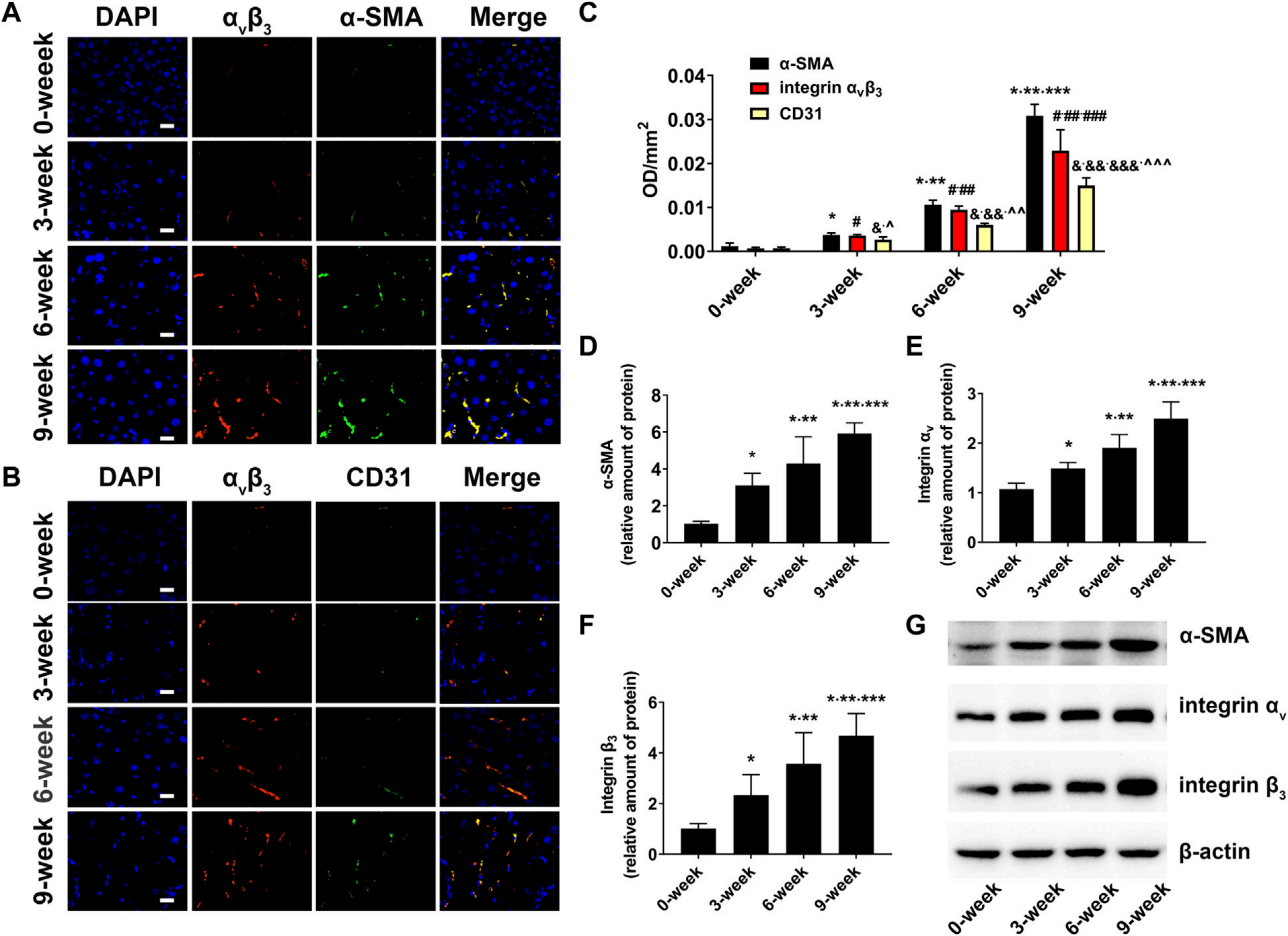
Biomarker expression in fibrotic livers **(A)** Immunofluorescent colocalisation of integrin α_v_β_3_ and α-SMA in the liver fibrosis groups and control group (×400; Scale: 20 μm). **(B)** Immunofluorescent colocalisation of integrin α_v_β_3_ and CD31 in the liver fibrosis groups and control group (×400; Scale: 20 μm). **(C)** Comparison of mean fluorescence densities of integrin α_v_β_3_, α-SMA and CD31 in the liver fibrosis groups and control group (*p < 0.05 versus 0-week group; **p < 0.05 versus 3-week group; ***p < 0.05 versus 6-week group; #*p* < 0.05 versus 0-week group; ##*p* < 0.05 versus 3-week group; ###*p* < 0.05 versus 6-week group; &*p* < 0.05 versus 0-week group; &&*p* < 0.05 versus 3-week group; &&&*p* < 0.05 versus 6-week group; ^*p* < 0.05 versus α-SMA in the 3-week group; ^^*p* < 0.05 versus α-SMA in the 6-week group); ^^^*p* < 0.05 versus α-SMA in the 9-week group). **(D–G)** Comparison of the protein level of α-SMA, α_v_ and β_3_ integrin subunits by western blot in the liver fibrosis groups and control groups (* *p* < 0.05 versus 0-week group; ** *p* < 0.05 versus 3-week group; *** *p* < 0.05 versus 6-week group).

To define the relationship between integrin α_v_β_3_, α-SMA, CD31, and liver fibrosis, immunofluorescence staining and western blotting were conducted. The immunofluorescence staining images and the corresponding average optical density results are presented in Figures 7A–C. As expected, only weak fluorescence was observed in the control group, but the fluorescence intensity and area started to increase in the CCl_4_-treated group from 3-week to 9-week, and the expression levels of α_v_β_3_, α-SMA, and CD31 were consistent with the progression of liver fibrosis. The results of the western blotting were in line with those of the immunofluorescence staining (Figures 7D–G).

Since the expression of integrin α_v_β_3_ is elevated upon both HSC activation and capillarisation of liver sinusoidal endothelial cells (LSECs) (Li, et al., 2011; Li, et al., 2016; Turaga, et al., 2021), overlay immunofluorescence staining of integrin α_v_β_3_ and α-SMA, integrin α_v_β_3_ and CD31 in fibrotic livers were performed to investigate the main factors of integrin α_v_β_3_ up-regulation. α-SMA is recognised as a specific marker of aHSCs (Wu, et al., 2019). In addition, CD31, a characteristic symbol of LSECs, was rarely expressed in normal liver tissues but positively expressed in fibrotic livers owing to sinusoidal remodelling and intrahepatic angiogenesis (Li, et al., 2011; Li, et al., 2016; Connolly, et al., 2010). In our study, the overlapped areas of integrin α_v_β_3_ and α-SMA were significantly larger than those of integrin α_v_β_3_ and CD31. This result was further supported by a higher average optical density value of α-SMA compared to CD31 in the fibrosis groups. Therefore, we assumed that aHSCs may be primary cells expressing integrin α_v_β_3_ during liver fibrosis, and the increased integrin α_v_β_3_ was more closely related to the activation of HSCs than neovascularization.

### 3.8 *In vivo* US/CT/MR imaging and image analysis

**FIGURE 8:**
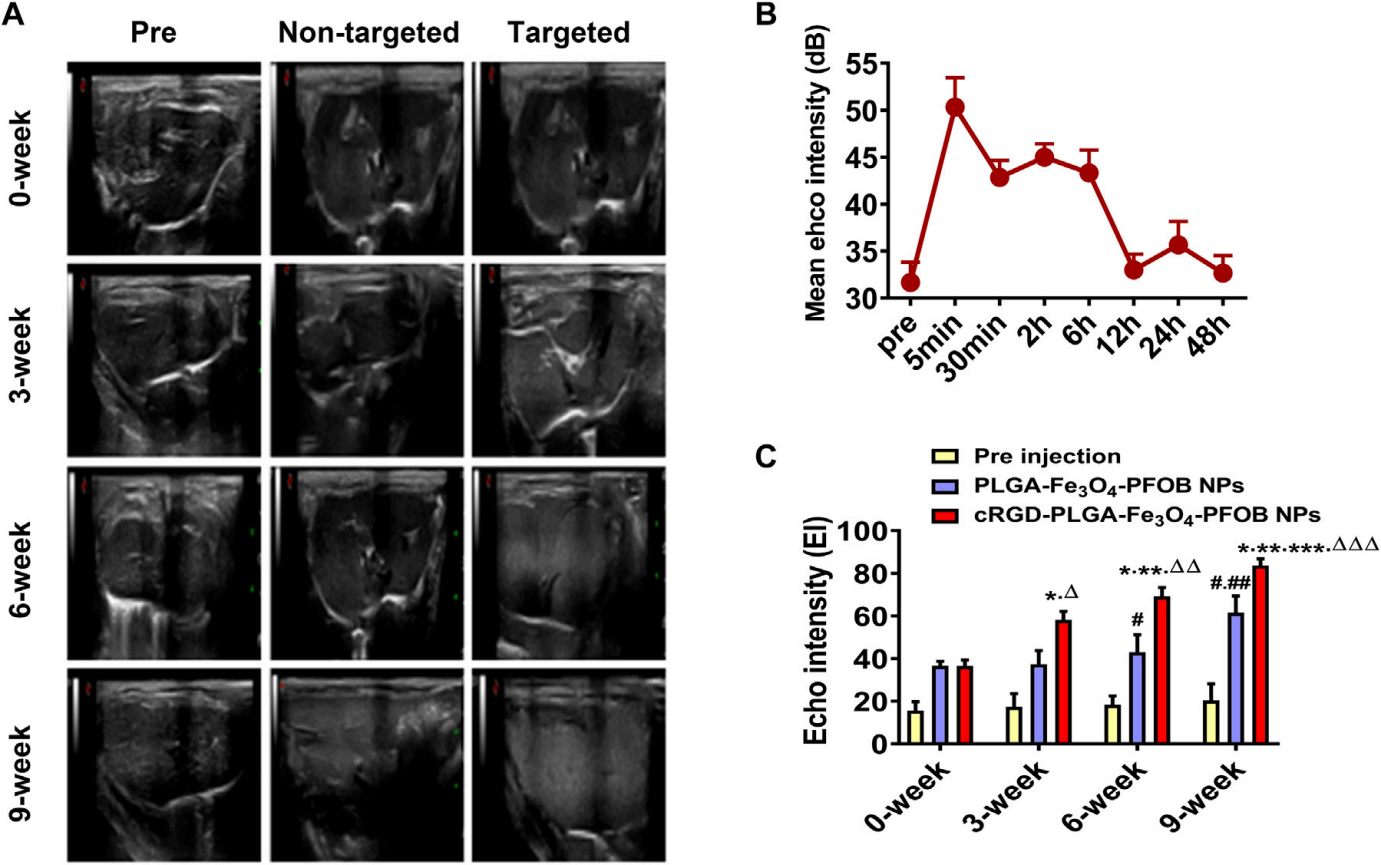
*In vivo* US imaging. **(A)** US images of liver before and after injection of targeted contrast agents (cRGD-PLGA-Fe_3_O_4_-PFOB NPs) or non-targeted contrast agents (PLGA-Fe_3_O_4_-PFOB NPs). **(B)** EI value of the liver parenchyma in the 0-week group within 48 h after injection of cRGD-PLGA-Fe_3_O_4_-PFOB NPs. **(C)** Comparison of liver EI values before and after injection of cRGD-PLGA-Fe_3_O_4_-PFOB NPs or PLGA-Fe_3_O_4_-PFOB NPs in each group (*p < 0.05 versus 0-week group, **p < 0.05 versus 3-week group, ***p < 0.05 versus 6-week group, #*p* < 0.05 versus 0-week or 3-week group, ##*p* < 0.05 versus 6-week group, Δ*P* <0.05 versus non-targeted 3-week group, ΔΔ*P* <0.05 versus non-targeted 6-week group, ΔΔΔ*P* <0.05 versus non-targeted 9-week group).

**FIGURE 9:**
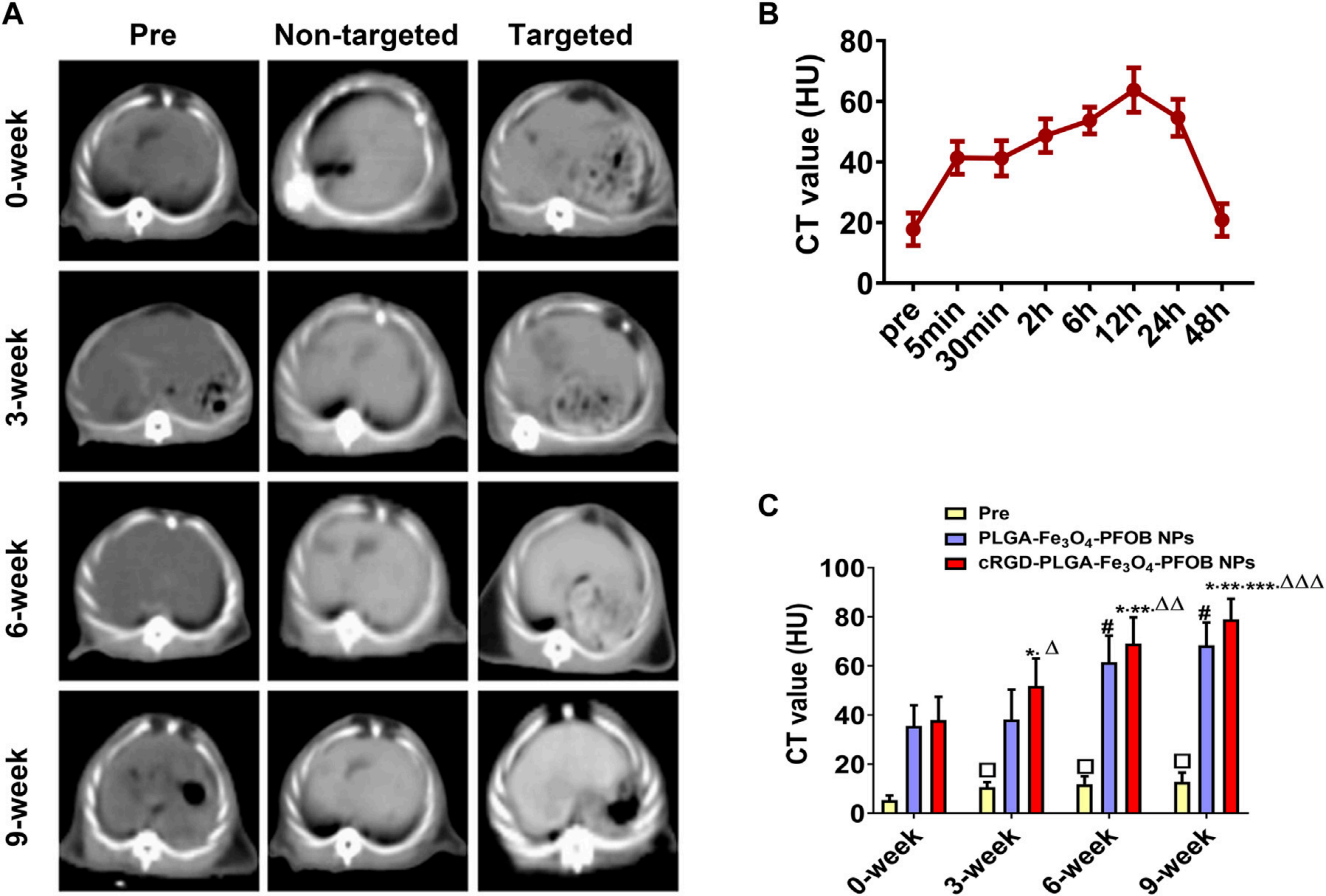
*In vivo* CT imaging. **(A)** CT images of liver before and after injection of targeted contrast agents (cRGD-PLGA-Fe_3_O_4_-PFOB NPs) or non-targeted contrast agents (PLGA-Fe_3_O_4_-PFOB NPs). **(B)** CT value of the liver parenchyma in the 0-week group within 48 h after injection of cRGD-PLGA-Fe_3_O_4_-PFOB NPs. **(C)** Comparison of liver CT values before and after injection of cRGD-PLGA-Fe_3_O_4_-PFOB NPs or PLGA-Fe_3_O_4_-PFOB NPs in each group (*p < 0.05 versus 0-week group, **p < 0.05 versus 3-week group, ***p < 0.05 versus 6-week group, #*p* < 0.05 versus 0-week or 3-week group, Δ*P* <0.05 versus non-targeted 3-week group, ΔΔ*P* <0.05 versus non-targeted 6-week group, ΔΔΔ*P* <0.05 versus non-targeted 9-week group, □*p* < 0.05 versus 0-week group).

**FIGURE 10:**
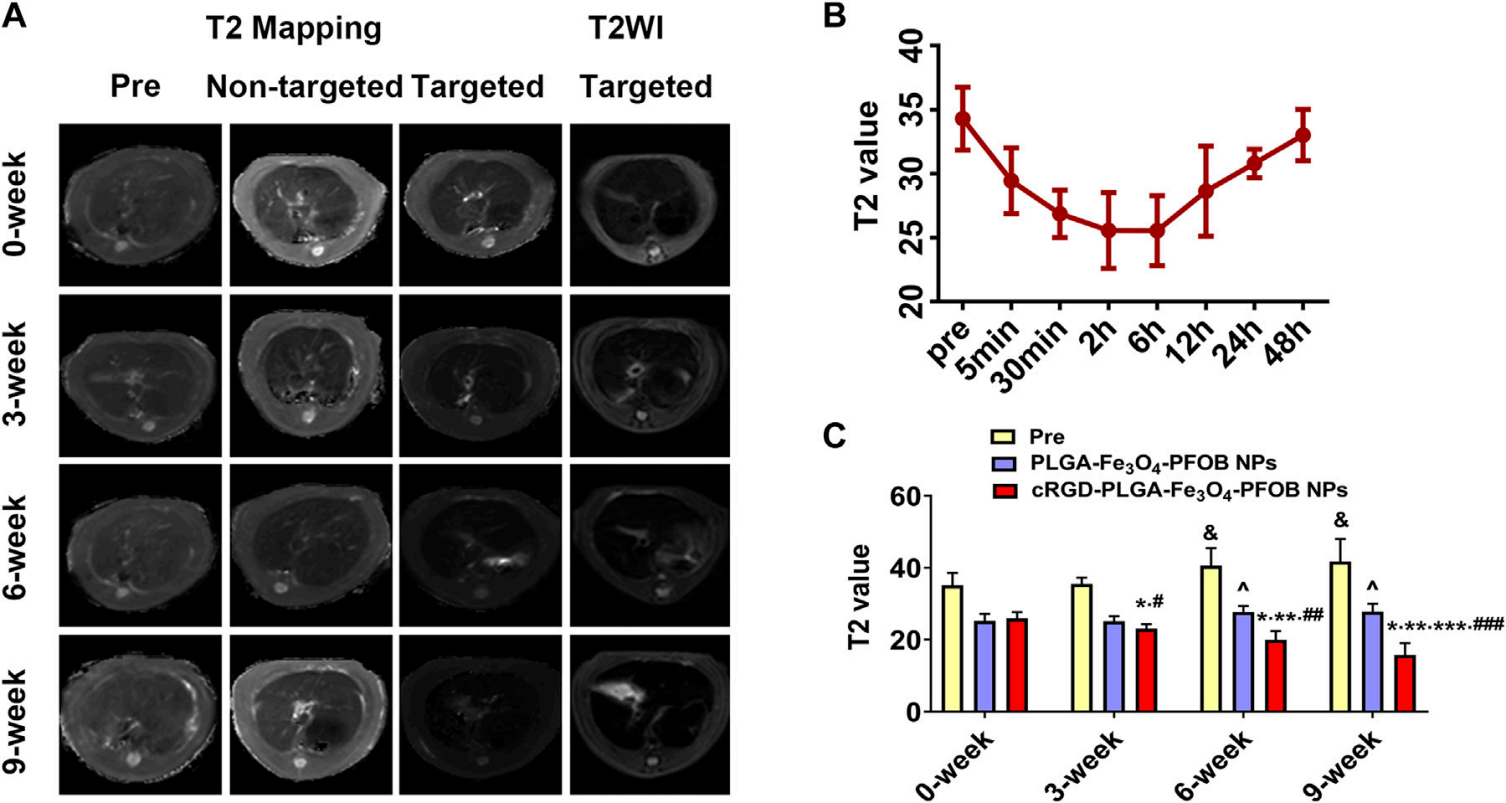
*In vivo* MR imaging. **(A)** MR images of liver before and after injection of targeted contrast agents (cRGD-PLGA-Fe_3_O_4_-PFOB NPs) or non-targeted contrast agents (PLGA-Fe_3_O_4_-PFOB NPs). **(B)** T2 value of the liver parenchyma in the 0-week group within 48 h after injection of cRGD-PLGA-Fe_3_O_4_-PFOB NPs. **(C)** Comparison of liver T2 values before and after injection of cRGD-PLGA-Fe_3_O_4_-PFOB NPs or PLGA-Fe_3_O_4_-PFOB NPs in each group (&*p* < 0.05 versus 0-week or 3-week group, ^*p* < 0.05 versus 0-week or 3-week group; *p < 0.05 versus 0-week group; **p < 0.05 versus 3-week group; ***p < 0.05 versus 6-week group; #*p* < 0.05 versus non-targeted 3-week group; ##*p* < 0.05 versus non-targeted 6-week group; ###*p* < 0.05 versus non-targeted 9-week group).

To further verify the effect of the cRGD-PLGA-Fe_3_O_4_-PFOB NPs on liver fibrosis staging *in vivo*, US, CT, and MR imaging were performed on the CCl_4_ rat models. All liver images before and 6 h after injection were captured and stored (Figures 8A, 9A, 10A). No significant differences in EI were observed among different fibrotic groups and the normal control group prior to NP injection. The CT values of the fibrosis groups were no statistical difference but higher than those of the normal group. Moreover, T2 values in the 6-week and 9-week groups were significantly higher than those in the 3-week group and 0-week group. Prior literature reporting T2 values measurements of human fibrotic liver samples corroborate our results, showing a stepwise increase in T2 values with the progression of fibrosis (Guimaraes, et al., 2016). A possible explanation presented by literatures is that an increased inflammatory component in chronic liver disease is related to the etiology of this finding (Oo, et al., 2010; Anstee, et al., 2010).

Early measurements after injection may be inaccurate because they reflect the concentration of NPs in the blood pool rather than those in the ECM. We thus first observed the changes in the quantitative index of liver parenchyma in normal rats at different time points after intravenous injection. The T2 values reached the peak level 6 h post-injection, indicating that the NPs reached the maximum accumulation level in the ECM. Although the CT values and EI values at this time were not the highest point, they were still maintained at a high level (Figures 8B, 9B, 10B). Therefore, to ensure that the enhanced effect of NPs could be maintained at a high level with sufficient targeting time, the observation and comparison time point at which subsequent imaging experiments should be performed, were determined to be 6 h post-injection. Similar to the results of previous studies (Zhang, et al., 2016; Xuan, et al., 2017; Wu, et al., 2019; Wang, et al., 2011), quantitative index comparisons showed the EI and CT values increased, while the T2 values decreased as fibrosis progressed following cRGD**-**PLGA**-**Fe_3_O_4_ **-**PFOB NPs injection (Figures 8C, 9C, 10C), which was indicative of greater accumulation of NPs due to the increased integrin α_v_β_3_ expression in liver. These imaging results suggested that cRGD**-**PLGA**-**Fe_3_O_4_ **-**PFOB NPs could act as novel tools to provide useful information for the identification of early liver fibrosis and noninvasive evaluation of liver fibrosis. An important advantage of this probe is that it combines independent biophysical properties measured by three imaging techniques, which offset the limitations of each measurement and generate broader and more feasible clinical application prospects.

Notably, both cRGD**-**PLGA**-**Fe_3_O_4_ **-**PFOB NPs and PLGA**-**Fe_3_O_4_ **-**PFOB NPs could be taken up by Kupffer cells, whereas cRGD**-**PLGA**-**Fe_3_O_4_ **-**PFOB NPs could also be specifically taken up by aHSCs in fibrotic livers (Wang, et al., 2011). To prevent possible accumulation of NPs in the Kupffer cells from affecting the measured results, we designed a control group of rats with an equivalent degree of fibrosis that was received equal doses of PLGA**-**Fe_3_O_4_ **-**PFOB NPs. Except for the normal group, the higher EI and CT values and lower T2 values were observed in rats injected with cRGD**-**PLGA-Fe_3_O_4_ **-**PFOB NPs compared to rats injected with PLGA**-**Fe_3_O_4_ **-**PFOB NPs, which was indicative of increased imaging effect due to the cRGD-medicated specific uptake by aHSCs. After the injection of PLGA**-**Fe_3_O_4_ **-**PFOB NPs, significant differences in EI and CT values between the 9-, 6-week group and the 0-, 3-week groups were observed. Then, the EI values of 9-week groups were higher than those of the 6-week groups. The increased accumulation of NPs can be attributed to the following factors: First, macrophages that increased with liver fibrosis progression may phagocytose more NPs (Wattacheril, et al., 2018). Second, angiogenesis and revascularization increased parallel with the severity of fibrosis (Xu, et al., 2010; Niu, et al., 2013). These newly formed LSECs are defective because of the wide fenestrations and the absence of smooth muscle layers. Third, the abnormal architecture often lacks effective lymphatic drainage. Although these three theories suggest lower T2 values in advanced fibrotic liver after the injection of PLGA**-**Fe_3_O_4_ **-**PFOB NPs, the T2 values of the 6- and 9-week groups were still higher than those of the 0-week and 3-week groups. We speculated that this phenomenon may be related to the smaller doses of NPs used in MR imaging than US and CT imaging and the limited encapsulation volume of Fe_3_O_4_ in PLGA, which resulted in an insufficient negative effect to offset the high T2 values in the 6- and 9-week groups prior to administration.

## 4 Conclusion

We showed expression levels of integrin α_v_β_3_ increased with fibrosis severity in CCl_4_ rat models, and the up-regulation level was more closely related to the activation of HSCs than neovascularization. In addition, our data demonstrated for the first time that combined US/CT/MR molecular imaging specifically target integrin α_v_β_3_ by using cRGD**-**PLGA**-**Fe_3_O_4_ **-**PFOB NPs was feasible for monitoring HSC activity and assessing liver fibrosis progression. The three techniques obtain complementary and different information with the potential to improve the diagnostic accuracy of a single imaging technique.

## Data Availability

The datasets presented in this study can be found in online repositories. The names of the repository/repositories and accession number(s) can be found in the article/Supplementary Material.
