# Supplementary material for: Enhanced US/CT/MR imaging of integrin αvβ3 for liver fibrosis staging in rat
Source: Front Chem. 2022 Oct 3;10:996116. doi: 10.3389/fchem.2022.996116 (PMC9574014; doi:10.3389/fchem.2022.996116)
Supplement: Supplementary file 1 [file DataSheet1.docx]

The dependence of traditional imaging techniques on liver morphological changes limits their accuracy in the diagnosis of early hepatic fibrosis. We employed PLGA to loaded Fe_3_O_4_ and PFOB then modified it with cRGD to obtain a targeted multimodal molecular contrast agent. These nanoparticles were small, less toxic, and could selectively target integrin α_v_β_3_-overexpressed hepatic stellate cells and serve as multimodal agents for staging liver fibrosis in vivo. Moreover, this study is the first to integrate ultrasound (US)/computed tomography (CT)/magnetic resonance (MR) triple-modality imaging methods for molecular imaging of liver fibrosis, which provides a broader and more feasible clinical application prospect than single imaging.
